# Association between phenotypic and genotypic characteristics and disease severity in individuals with cystic fibrosis

**DOI:** 10.1590/1984-0462/2023/41/2021286

**Published:** 2022-09-09

**Authors:** Gabriella Vieira Carneiro, Fabiana Sodré de Oliveira, Leandro Alves Pereira, Érica Rodrigues Mariano de Almeida Rezende, Luciana Carneiro Pereira Gonçalves, Vivian Mara Gonçalves de Oliveira Azevedo

**Affiliations:** a Universidade Federal de Uberlândia, Uberlândia, MG, Brazil.

**Keywords:** Cystic fibrosis, Genotype, Phenotype, Cystic fibrosis transmembrane conductance regulator, Fibrose cística, Genótipo, Fenótipo, Regulador de condutância transmembrana em fibrose cística

## Abstract

**Objective:**

To analyze the association between phenotypic and genotypic characteristics and disease severity in individuals with cystic fibrosis treated at a reference center in Minas Gerais, Brazil.

**Methods:**

This is a retrospective study that collected clinical and laboratory data, respiratory and gastrointestinal manifestations, type of treatment, Shwachman-Kulczycki score, and mutations from the patients’ medical records.

**Results:**

The sample included 50 participants aged one to 33 years, 50% of whom were female. Out of the one hundred alleles of the *Cystic Fibrosis Transmembrane Conductance Regulator* gene, the most prevalent mutations were DeltaF508 (45%) and S4X (18%). Mutation groups were only associated with pancreatic insufficiency (p=0.013) and not with disease severity (p=0.073). The latter presented an association with colonization by *Pseudomonas aeruginosa* and *Staphylococcus aureus* (p=0.007) and with underweight (p=0.036). Death was associated with age at diagnosis (p=0.016), respiratory symptomatology (p=0.013), colonization (p=0.024), underweight (p=0.017), and hospitalization (p=0.003).

**Conclusions:**

We could identify the association of mutations with pancreatic insufficiency; the association of *Staphylococcus aureus* colonization and underweight with disease severity; and the lack of association between mutations and disease severity. Environmental factors should be investigated more thoroughly since they seem to have an important effect on disease severity.

## RESUMO

**Objetivo:**

Analisar a associação entre as características fenotípicas, genotípicas e a gravidade da doença em indivíduos com fibrose cística atendidos em um centro de referência de Minas Gerais, Brasil.

**Métodos:**

Trata-se de um estudo retrospectivo, em que os dados clínicos, laboratoriais, as manifestações respiratórias e gastrointestinais, o tipo de tratamento, o escore de Shwachman-Kulczycki e as mutações foram coletados dos prontuários de registros dos pacientes.

**Resultados:**

A amostra incluiu 50 participantes, de um a 33 anos de idade, sendo 50% do sexo feminino. Do total de cem alelos do gene *Cystic Fibrosis Transmembrane Conductance Regulator*, as mutações mais prevalentes foram Delta F508 (45%) e S4X (18%). Os grupos de mutações apresentaram associação somente (p=0,013) com a insuficiência pancreática e não com a gravidade da doença (p=0,073). Esta última apresentou associação com a colonização por *Pseudomonas aeruginosa e Staphylococcus aureus* (p=0,007) e com baixo peso (p=0,036). O óbito foi associado com a idade no diagnóstico (p=0,016), a sintomatologia respiratória (p=0,013), a colonização (p=0,024), o baixo peso (p=0,017) e a ocorrência de internação (p=0,003).

**Conclusões:**

Foi possível observar associação entre as mutações e a presença de insuficiência pancreática; entre a colonização por *Staphylococcus aureus* e o baixo peso com a gravidade da doença; e ausência de associação entre as mutações e a gravidade da doença. Os fatores ambientais merecem ser investigados mais detalhadamente, pois parecem apresentar impacto importante na gravidade da doença.

## INTRODUCTION

Cystic fibrosis (CF) is an autosomal recessive disorder caused by mutations in the *Cystic Fibrosis Transmembrane Conductance Regulator (CFTR)* gene, located in the long arm of chromosome 7, which encodes the CFTR protein, important for maintaining electrolyte balance.^1^

The worldwide prevalence of CF is about 1:2,500 to 3,000, while in Brazil, it corresponds to 1:10,000 live births.^2,3^

More than 2 thousand *CFTR* gene mutations have been reported.^4^ They affect the synthesis and function of the CFTR protein, characterizing the disease as multisystemic.^1,5^ Mutations in the *CFTR* gene are divided into six functional classes. Usually, classes I, II, III, and VI cause loss of *CFTR* function or expression and are associated with more severe phenotypes. Classes IV and V reduce *CFTR* function or expression, causing mild to moderate disease phenotypes.^5^

The molecular analysis of the *CFTR* gene and the attempt to classify mutations into functional classes can optimize the treatment of affected individuals, contributing to identifying those eligible for specific mutation therapies, which act as CFTR correctors or potentiators.^6^ However, the correlation between genotype and phenotype has not been fully explained, given the heterogeneity of mutations, clinical manifestations, and other genetic and environmental factors involved in the modulation of this disease.^7^ Furthermore, the functional classification of mutations seems to have some limitations and should not be used alone to determine the relationship between genotype and phenotype.^7^

Considering this context, the present study aimed to analyze the association between phenotypic and genotypic characteristics and disease severity in individuals with CF treated at a reference center in Minas Gerais, Brazil.

## METHOD

This is a retrospective cross-sectional study. The research protocol was approved by the Human Research Ethics Committee of the institution, according to Resolution No. 466/12 of the Brazilian National Health Council (Opinion No. 3,669,592).

The study included individuals diagnosed with CF (confirmed by two sweat tests), followed by the multidisciplinary team of a reference center, who had a report of the molecular analysis of the *CFTR* gene. Out of 57 individuals, we excluded two because they did not have the report mentioned above; four who had no mutation justifying the clinical manifestation of CF; and one due to lack of information in their medical record.

Data were collected from the participants’ medical records between June and August 2020, and the findings were transcribed to a form elaborated for the study. Anthropometric and clinical characteristics were collected, as well as information on mutations in the *CFTR* gene. The variables age, weight, height, respiratory symptomatology, medications used, nutritional status, respiratory physiotherapy, unsupervised airway clearance therapy, and unsupervised physical activity were collected and analyzed considering the records of the last visit. For the variables age at diagnosis, history of hemoptysis, sinusitis, nasal polyps, meconium ileus, pancreatic insufficiency, hepatic fibrosis, hepatic steatosis, gallstones, gastritis, splenomegaly, hepatomegaly, kidney stones, diabetes, and mutation in the *CFTR* gene, we considered the first entries of each participant’s medical records (from birth to last entry). The colonization classification, the pathogen involved in the colonization, and hospitalization were collected based on entries from the previous year.

We analyzed the deaths that occurred in the two years prior to data collection, given the rarity of this event.

Disease severity was assessed using the Shwachman-Kulczycki score, which evaluates clinical characteristics such as overall activity, physical examination, nutrition, and radiological findings. Each of these categories receives a score of 05, 10, 15, 20, or 25. The final score represents the clinical manifestation, corresponding to the sum of the categories and classified as severe (<40), moderate (41–55), mild (56–70), good (71–85), or excellent (86–100).^8^ The Shwachman-Kulczycki score was obtained by calculating the mean score of the previous year for each individual. This score is not indicated to analyze disease severity in adults, especially with respect to nutritional status. However, we also considered the parameters weight-for-age, weight-for-height, height-for-age, and body mass index (BMI) for this assessment. The nutritional status of individuals under 18 years of age was classified according to the World Health Organization (WHO) growth curve .^9^

The laboratory data collected from medical records were: sputum cultures from the previous year (to identify the pathogens involved in the colonization); intermittent colonization, when less than 50% of cultures were positive for the same pathogen in the previous year; and chronic colonization, when more than 50% of cultures were positive for the same pathogen in the previous year; in addition to the molecular analysis of the *CFTR* gene.

Every individual has two alleles of this gene. Thus, the participants were categorized into four groups according to the most prevalent mutation, DeltaF508: DeltaF508/DeltaF508, DeltaF508/other mutation, other mutation/other mutation, and other mutation/absence of mutation. We chose this grouping, already used in previous studies,^10,11^ due to the high variability of these mutations in a small sample, in addition to limitations regarding the functional classification of *CFTR* gene mutations.^7^

Participants with only one mutation identified were not excluded from the study if the diagnosis was confirmed by the two sweat tests, with chloride values equal to or greater than 60mEq/L, and by the presence of at least one phenotypic characteristic of CF. Besides, the participants could have another mutated allele that was not identified by the adopted molecular analysis technique, as it used only exon capture followed by next-generation sequencing, not including the intron of the *CFTR* gene, which could identify other mutations.

We performed a descriptive analysis based on absolute and relative frequencies. Continuous variables (current age and age at diagnosis) were expressed as means and standard deviations or medians, categorical variables as absolute and relative frequencies, and the discrete variable (Shwachman-Kulczycki score) as means, medians, and standard deviations.

We used the Minitab software and the Poisson regression model to analyze the association between disease severity and the following phenotypic variables: respiratory symptomatology, current age, colonization classification, and nutritional status. Spearman’s correlation coefficient (Rho) was obtained to assess the relationship between age (numerical variable) and disease severity.^12^

The association of mutations (genotypic variables) with pancreatic insufficiency, respiratory symptomatology, and disease severity were analyzed using the Kruskal-Wallis test. This test was also employed to investigate the association of death with age, age at diagnosis, and other variables (gender, pancreatic insufficiency, colonization, respiratory symptomatology, nutritional status, supervised physiotherapy, and unsupervised physiotherapeutic interventions, such as airway clearance therapy, physical activities, and hospitalization).^12^

The R software was used for other analyses, considering p<0.05 as statistically significant.

## RESULTS

The study had a sample of 50 individuals aged one to 33 years. Among them, 25 (50%) were females, and 37 (74%) were Caucasians.

Only 28 (56%) of the participants presented positive neonatal screening, five (10%) were not screened, 15 (30%) presented negative screening, and two (4%) did not have this information in their medical records.

Table 1 presents the participants’ clinical and laboratory characteristics. The most prevalent age at diagnosis was the first month of life (44%), and most participants (n=23; 46%) presented an excellent Shwachman-Kulczycki score.

**Table 1.** Clinical and laboratory characteristics*.

Clinical and laboratory characteristics		n (%)	Clinical and laboratory characteristics		n (%)
Gender	Female	25 (50)	**Gastrointestinal manifestations**		
	Male	25 (50)	Meconium ileus		4 (8)
Ethnicity	Caucasian	37 (74)	Pancreatic insufficiency		45 (90)
	Not Caucasian	13 (26)	Hepatic steatosis		12 (24)
Age group	Infant	3 (6)	Hepatic fibrosis		1 (2)
	Preschool	11 (22)	Gallstones		6 (12)
	Schoolchildren	10 (20)	Gastritis		12 (24)
	Adolescent	15 (30)	Splenomegaly		2 (4)
	Adult	11 (22)	Hepatomegaly		2 (4)
**Diagnosis**			Nutritional status	Appropriate	34 (68)
Positive neonatal screening		28 (56)		Underweight/thin	23 (26)
Age at diagnosis	Up to 1 month	22 (44)	Overweight	3 (6)
	1 month to 2 years	18 (36)	**Other manifestations**		
	2 to 10 years	6 (12)	Kidney stones		3 (6)
	>10 years	4 (8)	Diabetes		3 (6)
**Disease severity**			**Treatments**		
Death		4 (8)	Pancreatic enzyme		45 (90)
Shwachman-Kulczycki score classification	Severe	1 (2)	Antibiotic		26 (52)
	Moderate	5 (10)	Inhaled glucocorticoids		26 (52)
	Mild	3 (6)	Mucolytics		40 (80)
	Good	18 (36)	Corticosteroid bronchodilator		28 (56)
	Excellent	23 (46)	Antiulcer agents		30 (60)
**Sinus and pulmonary manifestations**			Multivitamin supplement		48 (96)
Colonization classification	Lack of colonization	19 (38)	Ursodeoxycholic acid		9 (18)
	Intermittent colonization	13 (26)	Insulin		3 (6)
	Chronic colonization	18 (36)	Supervised physiotherapy		19 (38)
Pathogen (colonization)	*Pseudomonas aeruginosa*	7 (14)	Unsupervised airway clearance therapy		20 (40)
	*Staphylococcus aureus*	20 (40)	Unsupervised physical activity		25 (50)
	*Pseudomonas aeruginosa* and *Staphylococcus aureus*	4 (8)	**Exacerbation**		
	No colonization	19 (38)	Hospitalized in the previous year		14 (28)
Respiratory symptomatology (last visit)	Asymptomatic	9 (18)	Number of hospitalizations	1	4 (8)
	Typical symptoms	23 (46)		2	7 (14)
	Mildly exacerbated symptoms	11 (22)		≥3	3 (6)
	Moderately exacerbated symptoms	5 (10)			
	Severely exacerbated symptoms	2 (4)
Hemoptysis		2 (4)
Sinusitis		12 (24)
Nasal polyps		21 (42)

*Phenotype.

The most common treatments were multivitamin supplement (n=48; 96%) and pancreatic enzyme (n=45; 90%). Supervised physiotherapy and unsupervised physiotherapeutic interventions — airway clearance therapy and physical activity — were identified in 19 (38%), 20 (40%), and 25 (50%) individuals, respectively.

As for genotype, most participants were homozygous for the DeltaF508 mutation (n=15; 30%), followed by those heterozygous for the DeltaF508 mutation, with the S4X mutation in five (10%) participants (Table 2). All individuals have two alleles of the *CFTR* gene, totaling one hundred alleles in this sample. Among them, 45 (45%), 18 (18%), five (5%), and four (4%) presented DeltaF508, S4X, G542X, and R1162X mutations, respectively. Thus, functional classes I (27%) and II (45%) were the most prevalent.

**Table 2.** Prevalence of mutations in the *Cystic Fibrosis Transmembrane Conductance Regulator* gene and its functional classes.

Patients (%)	Allele 1 mutation			Allele 2 mutation		
	Traditional nomenclature	Current nomenclature HGVS (cDNA)	CF allele 1 mutation	Traditional nomenclature	Current nomenclature HGVS (cDNA)	CF allele 2 mutation
15 (30)	DeltaF508	c.1521_1523delCTT	II	DeltaF508	c.1521_1523delCTT	II
1 (2)	No nomenclature	c.1486delT	NC	DeltaF508	c.1521_1523delCTT	II
1 (2)	G542X	c.1624G>T	I	V317E	c.950 T>A	NC
2 (4)	5T	c.1210-12T[5]	V	Absence of mutation	Absence of mutation	Absence of mutation
2 (4)	S4X	c.11C>A	I	S4X	c.11C>A	I
1 (2)	R334W	c.1000C>T	IV	R1162X	c.3484 C>T	I
1 (2)	G542X	c.1624G>T	I	DeltaF508	c.1521_1523delCTT	II
1 (2)	2184insA	c.2052_2053insA	I	S4X	c.11C>A	I
1 (2)	S589N	c.1766G>A	III	DeltaF508	c.1521_1523delCTT	II
5 (10)	S4X	c.11C>A	I	DeltaF508	c.1521_1523delCTT	II
2 (4)	S4X	c.11C>A	I	G542X	c.1624G>T	I
1 (2)	G85E	c.254 G>A	II	R334W	c.1000C>T	IV
1 (2)	Exclusion of exon 2/CFTRdele2	c. (53+1_54-1) _ (164+1_165-1) del	I	DeltaF508	c.1521_1523delCTT	II
3 (6)	S4X	c.11C>A	I	R1162X	c.3484 C>T	I
1 (2)	G576A	c.1727 G>C	V	R668C	c.2002 C>T	III
1 (2)	R334W	c.1000C>T	IV	W1282X	c.3846G>A	I
1 (2)	1717-1G>A	c.1585-1 G>A	I	DeltaF508	c.1521_1523delCTT	II
1 (2)	No nomenclature	c.1505T>G	NC	Absence of mutation	Absence of mutation	Absence of mutation
1 (2)	S466X (TAG)	c.1397 C>G	I	DeltaF508	c.1521_1523delCTT	II
2 (4)	S4X	c.11C>A	I	3272-26A>G	c.3140-26A>G	V
1 (2)	A559T	c.1675 G>AT	II	DeltaF508	c.1521_1523delCTT	II
1 (2)	711+1G>T	c.579+1G>T	I	DeltaF508	c.1521_1523delCTT	II
1 (2)	S4X	c.11C>A	I	I507del	c.1519_1521delAT	II
1 (2)	S549R (T->G)	c.1647T>G	III	G542X	c.1624G>T	I
1 (2)	L206W	c.617T>G	IV	DeltaF508	c.1521_1523delCTT	II
1 (2)	R851X	c.2551C>T	NC	DeltaF508	c.1521_1523delCTT	II

FC: functional classification; NC: no classification; HGVS: Human Genome Variation Society.

DeltaF508 mutations in heterozygous individuals showed a significant association (p=0.013) with pancreatic insufficiency but not with disease severity (p=0.073) and respiratory symptomatology (p=0.666). After data analysis, the group with the combination other mutation/absence of mutation obtained better severity scores (median Shwachman-Kulczycki score of 95.0). The DeltaF508/other mutation combination was associated with worse Shwachman-Kulczycki scores (median=77.50), and all individuals with this mutation had pancreatic insufficiency.

Disease severity showed a significant association with colonization by *Pseudomonas aeruginosa* and *Staphylococcus aureus* (p=0.007) and the underweight nutritional status (p=0.036), that is, these characteristics contribute to disease severity, reducing the mean Shwachman-Kulczycki score (Table 3).

**Table 3.** Association between phenotype and disease severity.

Characteristics		Disease severity (Mean±standard deviation)	p-value
Respiratory symptomatology	Typical	85.9±9.3	*
	Exacerbated	74.1±17.5	0.071
Colonization	*Pseudomonas aeruginosa*	80.0±10.0	*
	*Staphylococcus aureus*	81.0±14.7	0.283
	*Pseudomonas aeruginosa* and *Staphylococcus aureus*	61.2±19.3	0.007
	No colonization	87.3±8.7	0.237
Nutritional status	Appropriate	86.9±6.4	*
	Underweight/thin	65.3±16.7	0.036
	Overweight	93.3±2.8	0.597
**Numerical variables**		**Rho**	**p-value**
Age		-0.165	0.099

*Reference values in the comparison by regression analysis.

Individuals with typical respiratory symptomatology presented a higher mean score than those with exacerbated symptomatology, that is, typical symptomatology was associated with lower disease severity.

Spearman’s correlation coefficient (Rho) indicated that younger individuals tended to have higher Shwachman-Kulczycki scores.

Death was significantly associated with age at diagnosis (p=0.016), as the younger the age at diagnosis, the lower the occurrence of death. In addition, the significant association of death with colonization revealed that individuals with colonization by *Pseudomonas aeruginosa* and *Staphylococcus aureus* had a higher risk of death. Exacerbated respiratory symptomatology (p=0.013), underweight (p=0.017), and hospitalization in the previous year (p=0.003) also showed a significant association with death (Table 4).

**Table 4.** Association between phenotype and death.

		Death		p-value
		Yes	No	
Age	n	4	46	0.158*
	Mean age (in years)	18.45	10.54	
Age at diagnosis	n	4	46	0.016*
	Mean age (in months)	47.00	2.00	
Gender	Female	3	22	0.608**
	Male	1	24	
Pancreatic insufficiency	Yes	4	41	0.491**
	No	0	5	
Pathogen (colonization)	*Pseudomonas aeruginosa*	1	6	0.024**
	*Staphylococcus aureus*	1	19	
	*Pseudomonas aeruginosa and Staphylococcus aureus*	2	2	
	No colonization	0	19	
Respiratory symptomatology	Exacerbated symptoms	4	14	0.013**
	Typical symptoms	0	32	
Nutritional status	Appropriate	0	34	0.017**
	Underweight/thin	4	9	
	Overweight	0	3	
Supervised physiotherapy	Yes	1	18	0.654**
	No	3	28	
Unsupervised airway clearance therapy	Yes	2	18	0.673**
	No	2	28	
Unsupervised physical activity	Yes	2	23	1**
	No	2	23	
Hospitalization	Yes	4	10	0.003**
	No	0	36	

*Kruskal-Wallis test for median comparison; **Kruskal-Wallis test for contingency tables.

## DISCUSSION

The present study found an association between *CFTR* gene mutations and pancreatic insufficiency, in addition to an association of disease severity with colonization by *Staphylococcus aureus* and *P. aeruginosa* and underweight.

Scientific evidence has shown^5,13,14^ that *CFTR* gene mutations are associated with pancreatic insufficiency but have a poor association with respiratory symptomatology. These findings may be related to the important influence of intracellular and physiological lung characteristics and environmental factors on pulmonary function, given the wide range of respiratory symptoms in individuals with CF.^5,13–16^ Environmental factors associated with pulmonary function in CF cases include the weather, air pollution, tobacco use, treatment adherence, access to care centers, and socioeconomic aspects.^15^

Although the present study did not analyze the association of the functional classification of mutations, other investigations^5,7^ showed that, usually, an allele with severe mutation belonging to functional classes I, II, III, or VI only leads to pancreatic insufficiency if paired with another allele with severe mutation. Therefore, one allele with mild mutation — class IV or V — preserves the pancreatic function even if combined with an allele with severe mutation since it presents residual Cl- channel activity in epithelial cell membranes.

*CFTR* gene mutations showed no significant association with disease severity. This result was similar to that of another study^10^ carried out in Brazil, which also analyzed disease severity in individuals with a mean age of 12.38±9.0 years, using the Shwachman-Kulczycki score. The research suggested a greater influence of other factors, such as gene modulation and poor treatment adherence, on the higher early mortality.^10^

Most study participants were homozygous (n=15; 30%) or heterozygous (n=15; 30%) for the DeltaF508 mutation, totaling 45 (45%) of one hundred alleles; this is the most prevalent and studied *CFTR* gene mutation in the world.^2–4,7,10^ It belongs to class II, meaning lack of functional CFTR protein and association with more severe CF phenotypes, with early respiratory symptoms, reduced pulmonary function, and pancreatic insufficiency.^5^ This result reinforces the influence of the environment on these patients’ prognosis, as the individuals investigated in this study showed no association between phenotypic and genotypic characteristics.

No association was found between respiratory symptomatology and disease severity, contrary to previous studies.^17,18,19^ This finding is probably due to the fact that the present study classified the respiratory manifestation based on clinical analysis, while other investigations used pulmonary function tests. In addition, most individuals were classified as asymptomatic (n=9; 18%) or presenting typical symptoms (n=23; 46%) regarding respiratory symptomatology, and, according to Stollar et al., the analysis of disease severity through the Shwachman-Kulczycki score may be limited when assessing patients with mild lung disease.^19^

The most prevalent pathogens were *Staphylococcus aureus* (n=20; 40%), *Pseudomonas aeruginosa* (n=7; 14%), and the combined colonization by *Staphylococcus aureus* and *Pseudomonas aeruginosa* (n=4; 8%). This finding was similar to that of recent studies, which identified a colonization change in European and American populations, with *Pseudomonas aeruginosa* as the most prevalent.^20,21^ However, a colonization that presented significant association with disease severity was the combination of *Staphylococcus aureus* and *Pseudomonas aeruginosa*, in contrast to a similar study, which detected an association between *Pseudomonas aeruginosa* colonization and CF severity.^17^ This fact may be related to the environmental differences between the sites where the studies were conducted. *Staphylococcus aureus* is the first pathogen to colonize individuals with CF, predisposing them, in adulthood, to *Pseudomonas aeruginosa* colonization, still considered the main responsible for progressive lung disease and, consequently, for the greater morbidity and mortality in most of these individuals.^16,22^

Disease severity also showed a significant association with nutritional status (underweight). According to previous studies, nutritional deficiency is associated with the severity of *CFTR* gene mutations, worse pulmonary function, exercise intolerance, and lower survival, as well as higher rates of chronic infections.^23,24,25,26^ This relationship can be justified by the fact that, in individuals with CF, underweight is caused by malabsorption — mainly due to pancreatic insufficiency — and higher energy expenditure related to excessive respiratory effort resulting from inflammatory conditions and pulmonary infections.^23,24^ Consequently, the reduced muscle mass lowers strength, respiratory muscle resistance, and exercise tolerance.^23,24,26^

Also, death showed a statistically significant association with age at diagnosis, colonization, respiratory symptomatology, nutritional status, and hospitalization. Late diagnosis is associated with a worse prognosis regarding pulmonary function, pancreatic insufficiency, nutritional status, and survival,^11,27,28^ in addition to higher rates of hospitalization and colonization.^28^ The implementation of neonatal screening is crucial for early diagnosis and the referral of these individuals to specialized centers without delay. Early follow-up improves the prognosis of the disease and reduces comorbidities.^28^

The association of death with colonization, respiratory symptomatology, nutritional status, and hospitalization can be explained by their direct or indirect relationship with respiratory complications, which are the main cause of mortality in CF.^22^

Although most participants did not undergo supervised physiotherapy and unsupervised physiotherapeutic interventions (airway clearance therapy and physical activity) — essential for a better disease prognosis^29^ —, these aspects showed no significant association with disease severity and death, a result that may be related to other variables not analyzed in the present study, such as frequency, type of exercises and physical activity practiced. The reference center where the study was conducted provides guidance for the practice of respiratory exercises and the importance of physical activity but does not have a specific physiotherapy program for patient rehabilitation. Usually, these patients are from other locations and receive physiotherapy in their city of origin. In addition to respiratory exercises, supervised physical activity has beneficial effects on pulmonary function, as well as anthropometric and biochemical parameters in individuals with CF.^29^

This study presents some limitations, such as the assessment of disease severity through the Shwachman-Kulczycki score, which is subjective. However, the participants are followed by the same professionals since the first visits, reducing the subjectivity of the score, which is the most used instrument to monitor CF severity.^19^ The score also presents a limitation related to the assessment of nutritional status in adults. In order to minimize this bias, we also considered the parameters weight-for-age, weight-for-height, height-for-age, and BMI.

The assessment of respiratory symptomatology presented limitations as well, as data were collected during a pandemic period, preventing us from obtaining recent respiratory function information. Additionally, we emphasize that, since we used a convenience sample, results such as the association between genotype and disease severity may be different in future studies performed with sample calculation.

In conclusion, *CFTR* gene mutations were significantly associated with pancreatic insufficiency but not with disease severity. Colonization and nutritional status showed a significant association with disease severity. Moreover, death was significantly associated with age at diagnosis, respiratory symptomatology, nutritional status, and hospitalization. These findings emphasize the importance of early diagnosis associated with the genetic analysis of individuals with CF to provide specific treatments. In addition, nutritional status needs to be carefully evaluated to favor the prognosis and survival of these individuals. Environmental factors should be investigated more thoroughly since they seem to have an important impact on disease severity, especially on respiratory function. We suggest the performance of prospective studies in the future.
